# Ultrasound-Guided Nerve Blocks for Painful Hand Injuries: A Randomized Control Trial

**DOI:** 10.7759/cureus.18978

**Published:** 2021-10-22

**Authors:** Michael Vrablik, Arvin Akhavan, David Murphy, Caitlin Schrepel, Michael K Hall

**Affiliations:** 1 Department of Emergency Medicine, University of Washington, Seattle, USA

**Keywords:** ultrasound, nerve block, forearm, acute pain, pain managment

## Abstract

Objectives: Traumatic hand injuries present to emergency departments frequently. Pain secondary to these injuries is typically managed with opioids, which may be inadequate and have side effects. Ultrasound (US)-guided forearm nerve blocks have emerged as an alternative modality for patients with acute pain from isolated extremity injuries.

Methods: We performed a non-blinded, consecutive, randomized pragmatic trial of US-guided forearm nerve blocks using medium and long-acting anesthetic versus usual care for a six-day period around July 4th, 2017. Adults who sustained a traumatic or blast injury of their hands were considered. Consecutive emergency department patients were consented, enrolled and randomized into a study group (block) or control (standard care). The study group received a US-guided forearm block using a 50/50 mix of 1% lidocaine and 0.5% bupivacaine. The primary outcome was median pain scores via a 100-point visual analog scale at 15, 60, and 120 minutes after the nerve block compared to the baseline pain score. The secondary outcome was mean morphine equivalents administered.

Results: Sixteen patients were screened and 12 were randomized: six to the treatment group and six to the control group. Median pain reduction from baseline at 15, 60, and 120 minutes in the forearm block group was -35 (IQR=10), -30 (IQR=50), and -20 (IQR=70, versus -5 (IQR=10), -20.5 (IQR=20), -20 (IQR=70) in the control group. At all time points, patient-reported pain scores decreased significantly over baseline in the forearm block group, whereas non-significant reductions in pain scores occurred in the control group.

Conclusion: US-guided forearm blocks for acute traumatic hand injuries resulted in greater pain relief when compared to usual care.

## Introduction

Hand and forearm pain secondary to fracture, laceration, dislocation, infection, and blast injury is a common issue in the emergency department (ED) [1]. In the United States, a significant increase in the number of firework-related injuries occurs in the weeks around July 4 (Independence Day), and hand injuries are the most common firework-related injury [2]. In 2017, there were 12,900 ED visits for firework-related injuries in the United States, with 8,700 of those injuries occurring within one month period from June 16 to July 16. Of these patients that presented to the ED, the most common injuries were to the hand and fingers [2]. Achieving adequate analgesia is challenging considering the dense sensory innervation of the hand [3], unique injury patterns, and the need for manipulation of the injured extremity to facilitate examination, splinting, reduction, wound exploration, and closure. Traditional ED pain management centers on parenteral opioid and non-opioid analgesics; however, these may be limited by drug availability [4,5] adverse effect profiles, and efficacy [6]. Importantly, opiate naive patients who are prescribed even a small number of opiates at discharge may be at increased risk of addiction [7], and many emergency departments are exploring non-opioid options for acute pain control.

In a recent publication, 121 of 171 emergency medicine residencies provided information on the usage of regional anesthesia in the ED with 84% of programs performing US-guided nerve blocks, most commonly forearm nerve blocks (ulnar, median, or radial nerves). Nerve block techniques are taught via didactic sessions, online resources, and supervised training. Most residency programs do not have specific agreements with other specialty services with regard to performing US-guided nerve blocks in the ED [8]. An interdisciplinary approach between emergency medicine, orthopedics, and anesthesiology resulted in a case series where patients’ pain was controlled well with US-guided nerve blocks and thus allowed proper evaluation of the injuries, irrigation of the wound thoroughly, and employment of temporizing measures such as sutures and splints while the patient waited for definitive management. Furthermore, there were no cases of compartment syndrome [9].

## Materials and methods

This study was reviewed and approved (STUDY00001627) by the Institutional Review Board for Human Subjects Research at the University of Washington. Prior to the study, a multidisciplinary group consisting of emergency medicine, orthopedic surgery, and plastic surgery formalized a forearm block protocol. The protocol outlined four key components that would guide patient selection, procedural readiness, and patient safety. First, the hand surgery consultant would provide a timely evaluation of the patient in the ED and perform a standardized hand examination to discern if and when surgery was needed. Second, the ED provider administering the nerve block would reference weight-based local anesthetic dosing to ensure appropriate and safe dosing. Third, we created a “block box” that held all the necessary tools (i.e., sterile gel, sterile probe covers, syringes, needles, tubing) for the ED provider to have at the patient bedside for the procedure. Finally, we ensured immediate access to lipid emulsion should there be any complication resulting in local anesthetic toxicity.

Study design and participants

**Figure 1:**
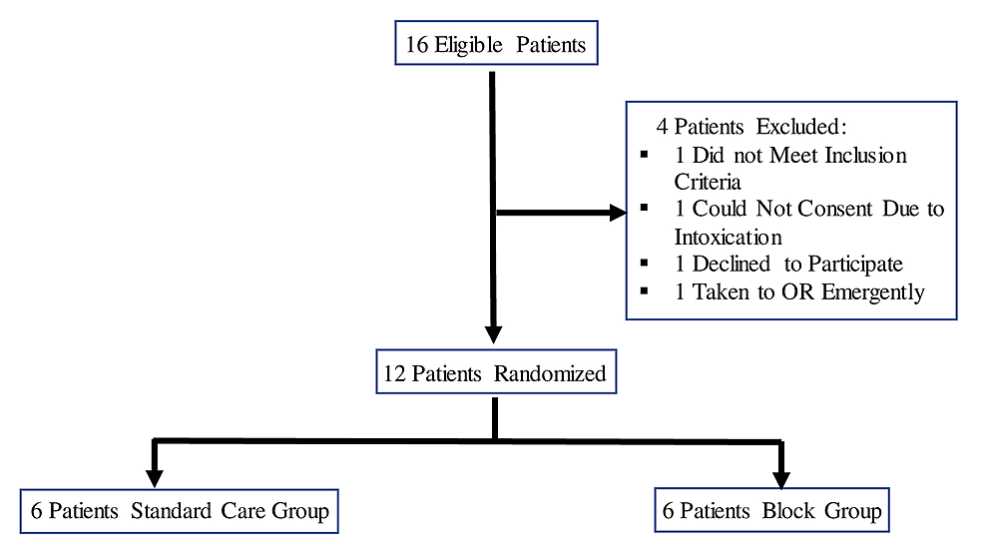
Enrollment and randomization flow diagram

This study was conducted at an urban, academic, Level I trauma and burn center with an average of 66,000 annual ED visits. We consecutively enrolled consenting adult patients with trauma to the hand presenting to an academic, Level I trauma and burn center, between July 1 and July 6, 2017. Potential patients were approached consecutively by the study team and were included if they were: 18 years or older, clinically stable, sustained an injury to the upper extremity injury distal to the wrist requiring pain control, and were able to consent. Subjects were excluded if they were: at high risk for compartment syndrome as determined by the primary ED physicians, immediately going to the operating room, requiring emergent interventions for polytrauma, unable to be consented (e.g., due to intubation or clinical intoxication), or incarcerated. Once consent was obtained, the patient was randomized to the treatment arm (nerve block + usual care) or the control arm (usual care). The patient received a standardized and detailed hand examination by the hand surgery consultant. If randomized to the treatment arm, the patients were then eligible for US-guided forearm nerve block to be performed by a trained emergency medicine physician study team member (attending or senior resident) in addition to pain management as determined by the primary ED treating team. All study team members had completed training including a didactic session as well as practice performing simulated blocks on a phantom model. A review of the EHR at 30 days after enrollment was performed to assess for complications. This study was performed in a manner consistent with CONSORT criteria (Figure 1) [10].

Nerve block Procedure

**Figure 2:**
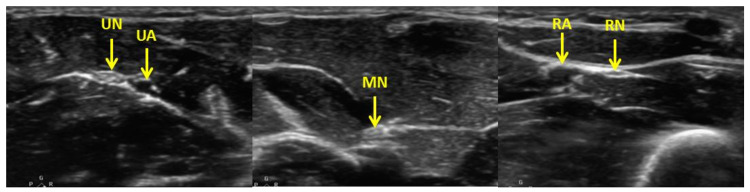
US images of the ulnar nerve (UN) in relationship to the ulnar artery (UA), median nerve (MN), and radial nerve (RN) in relation to the radial artery (RA).

The patient received US-guided forearm nerve blocks of the radial, median, and ulnar nerves depending on the pattern of injury (Figure 2).

A sparing approach was taken such that only the nerves innervating the injured area were blocked to minimize the risk of peripheral nerve injury from the block (e.g., isolated injuries to the small finger and fifth metacarpal might only receive an ulnar nerve block). The patient’s forearm was prepped with chlorhexidine and draped in an appropriate sterile fashion. Using a high frequency, linear transducer with a SonoSite X-Porte (FujiFilm SonoSite Inc., Bothell, WA) fitted with a sterile probe cover, the median, radial, and ulnar nerves were identified in cross-section with ultrasound (US). Using an in-plane needle approach, a 20-gauge spinal needle attached to a 10mL syringe was advanced toward the nerve. Three to five milliliters of a 50/50 mix of 1% lidocaine and 0.5% bupivacaine was infiltrated around the nerve, with care taken not to inject within the nerve. If necessary, standard pain management was allowed for the intervention group per the primary clinical care team.

Pain assessment

Following randomization, the patient-reported pain scores were recorded (prior to intervention in the nerve block group). Among patients who had bilateral injuries, one side was randomly selected for analysis of pain scores and recorded. Pain scores were assessed independently using a visual analog slider with a scale ranging from 0-100 provided by REDCap (REDCap, Nashville, TN) at standardized times of 0 (prior to the procedure), and then 15, 60, and 120 minutes after completion of a nerve block (or randomization in the control group). If the patient was unable to use the slider because of their injuries, they would verbally instruct a family member/friend to move the slider on the scale to reflect their current pain severity.

Outcomes

The primary outcome was median pain scores via a 100-point visual analog scale at 15, 60, and 120 minutes after the nerve block compared to the baseline pain score. The secondary outcome was mean morphine equivalents administered.

Statistical analysis

A Wilcoxon signed-rank test was performed comparing median VAS scores before and after the nerve blocks for each patient. All analyses were performed using Stata 14.2 (Stata Corporation, College Station, TX). A power calculation for the 15-minute group comparison, where we expect to see the largest difference, including the power of 80%, and alpha set at 0.05 was performed prior to enrollment using estimation for a clinically significant reduction in pain severity in accordance with prior studies [11,12]. Using an estimated 13-20mm VAS change in pain severity between groups as a minimum clinically important difference, and standard deviation of 10-15 mm for VAS, indicating that we will need to include 12-20 total subjects.

## Results

Characteristics of the study cohort

Sixteen patients were approached and screened for enrollment into the study during the dates July 1 to July 6, 2017. Of these 16 patients, four were not able to be enrolled. Of these four patients, two did not meet inclusion criteria (one patient was taken to the OR and one patient was too intoxicated to consent) and two declined to participate (one patient declined because they did not have pain and the other did not want to be part of the study). A total of 12 patients were enrolled and randomized to the treatment group (N=6) and the control group (N=6). The median age of all enrolled patients was 41 years (19-72) and all were male. Among both groups, nearly all patients arrived by emergency medical services, and the majority of injuries were resultant from a blast mechanism (Table 1).

**Table 1:** Characteristics of the participants

		Control	Intervention - US Nerve block
N		6	6
Gender	Male	6	6
Mean Age		34.5	47.3
Arrival Method	Aeromedical Transport	0 (0%)	1 (17%)
	Advanced Life Support Ambulance	4 (67%)	1 (17%)
	Basic Life Support Ambulance	1 (17%)	3 (50%)
	Self-Presentation	1 (17%)	1 (17%)
EtOH consumption?	Yes	2 (33%)	1 (17%)
Hand Side(s)	Bilateral	3 (50%)	2 (33%)
	Left	0 (0%)	1 (17%)
	Right	3 (50%)	3 (50%)
Injury mechanism	Blast	4 (67%)	3 (50%)
	Burn	0 (0%)	1 (17%)
	Grinder	1 (17%)	1 (17%)
	Other	1 (17%)	1 (17%)

Among those randomized in the treatment group, a total of 22 nerve blocks were performed (eight radial, eight median, and six ulnar). All blocks were performed by study team members.

Main results

Median pain reductions in VAS at 15, 60, and 120 minutes after nerve block in the intervention group were -35 (IQR=10), -30 (IQR=50), and -20 (IQR=70), on a 100-point scale compared to baseline. In the control group, median pain reductions in VAS at 15, 60, and 120 minutes after randomization were -5 (IQR=10), -20.5 (IQR=20), -20 (IQR=70) compared to baseline (Table 2).

**Table 2:** Reduction in pain score vs. baseline

Time after the initial block	Control group	Significance	Nerve block group	Significance
	Median (IQR)	P-value	Median (IQR)	P-value
15m	-5 (-10, 0)	0.39	-35 (-40, -30)	0.03
1h	-20.5 (-30, -10)	0.06	-30 (-50, 0)	0.05
2h	-20 (-70, 0)	0.09	-20 (-30, -10)	0.04

Patient-reported pain decreased, compared to baseline, in the intervention group at 15 and 120 minutes (p<0.05), and non-significant reduction at 60 minutes (p=0.05). One patient in the group reported continued paresthesia at three days post-intervention. No patients had paresthesias at 30 days. No other significant complications were reported.

Secondary results

MME administered for the intervention group was 6mg (IQU 2-12mg) compared to the 13mg in the control group (IQR4/16mg) with a p-value of 0.58. While the control group did receive more IV opioids there was no statistical significance when comparing the two groups.

## Discussion

This small randomized trial of patients undergoing US-guided forearm nerve blocks for acute traumatic hand injuries found significant improvement in patient-reported pain and 15 minutes and 120 minutes after the procedure, as compared with non-significant reductions in pain in the control group. The demographics, mode of arrival, mechanism of injury, and injury patterns were similar among the two groups. Fewer IV opioids were administered among the intervention group, although there was no statistical difference.

There were no reported adverse events related to the nerve blocks. Collectively, these findings suggest US-guided forearm blocks are feasible and may be an efficacious and safe modality to relieve pain for acute traumatic hand injuries and limit opioid use.

Regional nerve blocks have proven a useful tool in the management of extremity pain, and the use of direct visualization with US can minimize risks and provide improved anesthesia of the hand when compared to the landmark-based approaches [13-16]. Studies measuring feasibility have found that these blocks can be done in less than 10 minutes, and without significant complications [12,17]. Recent emergency medicine literature has demonstrated the successful use of US-guided regional nerve blocks in the ED for finger reduction [18], upper extremity fractures, dislocations, abscess drainage [17], and hand blast injuries [9]. Widespread adoption of US-guided nerve blocks can add to a larger portfolio of multimodal analgesia and limit exposure to opioids. However, prior to implementing the use of regional nerve blocks in ED for acute traumatic pain, a collaborative, multidisciplinary framework should first be established for patients requiring surgical consultation. The current study demonstrates a possible protocol for implementation. Also, this study shows the efficacy of US-guided forearm nerve blocks in pain reduction at a busy, urban, Level I trauma center.

This study has limitations. First, the total number of patients that met inclusion criteria was small. This was, in part, due to a narrow study design in order to limit the confounding effects of other major painful trauma to other organ systems. Further, the study procedure must not interfere with critically ill or potentially life-saving interventions being provided to polytrauma patients. Second, the study was not blinded to patients, investigators, or the primary clinical care team. Future studies could utilize sham blocks, and effectively blind all parties to the administration of regional anesthetic. Third, outcome reviews were limited to the hospital system in which the patient presented for study enrollment, and therefore may have missed complications diagnosed at outside hospital visits. Finally, it is possible that patients who had more severe injuries might have biased the results; however, we controlled for bilateral injuries by random selection of laterality for pain assessment, as well as performing the analysis of pain reduction between specific subjects as opposed to comparing group means or medians to account for the variability of how individual persons experience and report pain.

## Conclusions

US-guided nerve blocks have been shown to be an effective pain management modality in the emergency department, specifically, the use of US guided-forearm blocks. These forearm blocks provide the ED physician another tool to manage patients with severe pain in the acute period, such as hand blast injuries. Our study demonstrates that a US-guided nerve block of the forearm gives greater pain control earlier in the course of the patient’s care in comparison to traditional intravenous therapy alone. Early regional anesthesia among patients with hand injuries in ED appears to be effective, feasible, safe and may result in lower opioid use.

Additional studies in this area would be beneficial. Studying a larger cohort of patients with painful hand injuries and utilizing a blinded approach (i.e., placebo nerve blocks) may provide further support for the use of US-guided forearm blocks for painful hand injuries.
